# Re-vitrectomy for recurrent retinal detachment in post-vitrectomy eyes of rhegmatogenous retinal detachment

**DOI:** 10.1186/s12886-022-02665-8

**Published:** 2022-11-16

**Authors:** Jun-Xing Bai, Wei-Yu Zheng, Xiao-Qing Zhu, Xiao-Yan Peng

**Affiliations:** 1 Beijing Ophthalmolgy and Visual Science Key Laboratory, Department of Ophthalmology, Beijing Tongren Hospital, Capital Medical University, No.1 Dongjiaomin Alley, Dongcheng District, Beijing, 100730 People’s Republic of China; 2 Department of Ophthalmology, Beijing Hua-Er Hospital, No.59 Xizongbu Lane,Dongcheng District, Beijing, 100005 People’s Republic of China; 3 Department of Ophthalmology, Beijing Meiermu Hospital, No.65 Fuxing Road, Haidian District, Beijing, 100036 People’s Republic of China

**Keywords:** Recurrent retinal detachment, Pars plana vitrectomy, Re-vitrectomy, Post-vitrectomy eye, Contralateral eye

## Abstract

**Background:**

Recurrent retinal detachment (Re-RD) usually affects the prognosis of surgery for rhegmatogenous retinal detachment (RRD). Previous clinical studies of Re-RD were not specific. This study aimed to analyze the clinical characteristics of Re-RD in post-vitrectomy eyes with RRD and surgical outcomes after revitrectomy without combining it with retinectomy or scleral buckling.

**Methods:**

This is a retrospective case series analyzed the ocular characteristics of 20 recurrent and contralateral eyes, evaluated the significance of the associations between variables before reoperation and the final best-corrected visual acuity (BCVA), and calculated the outcome of revitrectomy.

**Results:**

Patients with phakic eyes, those undergoing only one surgery, and those with more than one break had better final BCVA. The final BCVA was negatively correlated with the axial length and positively correlated with the preoperative BCVA. Among the 12 eyes with no break detected before surgery, 11 (92%) were found to have a small crevice-like break beside the pigment scar of a large number of original laser spots. The single-operation complete retinal reattachment rate was 75%, the complete retinal reattachment rate was 80%, and the final incomplete retinal reattachment rate was 90%. The BCVA improved from 1.2 ± 0.6LogMAR (0.06 ± 0.25) before surgery to 0.8 ± 0.7LogMAR (0.15 ± 0.2) at the last follow-up. The BCVA of 16 patients with complete retinal reattachment improved from 1.0 ± 0.5LogMAR (0.1 ± 0.3) to 0.6 ± 0.4LogMAR (0.25 ± 0.4). In the contralateral eyes, 15% already had vision-damaging disease, and the incidence of eyesight-threating lesions was 5.9% during follow-up.

**Conclusions:**

Revitrectomy without retinectomy or scleral buckling can effectively treat Re-RD in post-vitrectomy eyes. In Re-RD patients with no definite retinal break detected preoperatively, the retinal hole usually shows small crevice-like changes alongside a large number of original laser pigment scars.

## Background

Recurrent retinal detachment (Re-RD) is one of the most common reasons affecting the prognosis of surgery for rhegmatogenous retinal detachment (RRD). Reports on the treatment and prognosis of Re-RD are relatively fewer than those of RRD in the past [1–8]. Previous clinical studies on Re-RD had the several disadvantages. (1) In many articles, patients with various etiologies, such as RRD, vascular diseases, or penetrating injury, were included in the analysis of the characteristics of Re-RD [1–3, 6]. (2) Although some authors uniformly included patients who had Re-RD after surgery for RRD, the primary surgical methods in the same study were different, including scleral buckling, vitrectomy, or retinopexy [9–11]. (3) Even though some studies evaluated the effectiveness of reoperation for Re-RD patients according to the initial surgical method respectively, the vitreous status of Re-RD patients after vitrectomy was different, including both silicone oil filled or no tamponade eyes [12]. Although such research can provide a certain understanding of the overall situation and prognosis of Re-RD, it is not of great clinical significance or reference value for a certain group of people.

Re-RD after vitrectomy with gas tamponade or vitrectomy with silicone oil removal (vitrectomy) progresses rapidly and may easily lead to proliferative vitreoretinopathy (PVR). However, this treatment remains challenging. There have been a few reports on the treatment of Re-RD after vitrectomy for RRD, and the surgical methods are relatively radical, retinectomy for all [10], combined with scleral buckling for all [13], or retinectomy combined with scleral buckling [14]. However, the complications of low intraocular pressure (IOP) and silicon oil (SO) dependence after retinectomy are not friendly for the long-term prognosis of patients [10, 15, 16]. Increased myopia and astigmatism, prolonged operation time, and aggravated post-surgical inflammation caused by combined scleral buckling may also lead to poor prognosis [13, 14, 17, 18].

This study aimed to analyze the characteristics of Re-RD and to report the anatomical reattachment rate and visual acuity recovery after revitrectomy without retinectomy or scleral buckling for Re-RD in post-vitrectomy eyes with RRD. We aim to provide a thorough understanding of Re-RD and clinical evidence for developing the best treatment plan for this specific group of patients.

## Methods

### Participants

Surgeries were performed after obtaining informed consent from all patients. This study adhered to the tenets of the Declaration of Helsinki. Data were obtained retrospectively by reviewing the medical records of post-vitrectomy eyes that underwent pars plana vitrectomy (PPV) between January 2017 and December 2021 to treat Re-RD. Only eyes previously diagnosed with RRD that underwent PPV at least once were included in the study. The exclusion criteria were secondary RRD resulting from trauma, endophthalmitis, or vascular disease such as diabetic retinopathy; previous treatment with scleral buckling; Re-RD with silicone oil in situ; Re-RD due to macular hole; and follow-up time < 3 months. Twenty consecutive eyes were included in the study. Of the 20 patients, 12 underwent primary PPV surgery for RRD by Dr. X.Q.Z., and 8 were referred for further management after undergoing one or more unsuccessful PPV surgeries by other surgeons.

### Surgical techniques

The surgical procedures were performed under general anesthesia by the same surgeon (Dr. X.Q.Z.). A standard three-port 23-gauge PPV approach was used in all the cases. All operations were performed on an Alcon Constellation (Alcon Laboratories, USA) with the assistance of non-contact widefield viewing systems for visualization (RESIGHT®700, Carl Zeiss, Germany).

Triamcinolone acetonide suspension was used in every patient to visualize the residual vitreous and ensure that posterior vitreous detachment was accomplished in every patient. Indocyanine green staining was used when necessary in identifying or assisting in the removal of pre-retinal membranes with forceps. The peripheral vitreous base was shaved as cleanly as possible through the indentation of the anterior sclera with the help of an assistant. During vitrectomy, perfluorocarbon liquid was used as required. Laser retinopexy was applied in a confluent two-to-three row fashion around all retinal breaks. An intraocular tamponade agent of either non-expansile gas concentration (20% SF6 or 14% C3F8) or silicone oil (5700 centistokes [cS]) was utilized according to the patient’s condition and the surgeon’s experience. Phacoemulsification was not routinely performed in phakic eyes and was performed only if necessary because of severe opacification obstructing the surgeon’s view of the posterior segment. Patients with gas tamponade were asked to put their face down for 2–4 weeks, and patients with SO tamponade were asked to put their face down for 4 weeks after surgery.

### Data collection

All eyes underwent a complete ocular examination, including a slit-lamp examination and binocular indirect ophthalmoscopy. The preoperative data collected included the patient’s age, sex, laterality of the eye, past surgical history, duration of Re-RD, lens status, number and location of breaks, extent of Re-RD (quadrant), macular status (on or off), PVR grading (Retina Society Terminology Committee Classification 1983), axial length, best-corrected visual acuity (BCVA) at baseline, and IOP measured by noncontact tonometry. Data obtained from the surgical records identified the localization and number of retinal breaks, intraoperative complications, and type of tamponade. Postoperative data included BCVA, IOP, length of follow-up, postoperative complications, additional surgical procedures, presence of SO, retina status, and contralateral eye condition.

Retinal status was defined according to anatomic success and the presence of SO as follows (PPV + SO and SO removal were defined as a single operation):

1. Recurrent retinal detachment: The retina was detached at the last follow-up with or without SO in the vitreous.
2. Macular attachment: the macula is attached with or without SO in the vitreous.
3. Incomplete retinal attachment: the retina was attached at the last follow-up, with or without SO in the vitreous.
4. Complete retinal attachment: the retina was completely attached at the last follow-up, without SO in the vitreous.
5. Single-operation complete retinal attachment: the retina is completely attached by a single operation at the last follow-up without SO in the vitreous.

The primary outcome was the characteristics of Re-RD, retinal status, and BCVA at the last follow-up. Other outcome measures included postoperative complications and contralateral eye conditions.

### Statistical analysis

The Snellen’s visual acuity was converted to the logarithm of the minimum angle of resolution (logMAR) equivalent for analysis. The logMAR denotations for non-numeric visual acuities were: counting fingers = 1.7 logMAR; hand motion = 2.0 logMAR; light perception = 2.3 logMAR; and no light perception = 3.0 logMAR [5]. Statistical analysis was performed using SPSS (version 26, IBM SPSS statistics). Continuous variables are expressed as mean ± SD, and categorical variables are expressed as individual counts and proportions. The significance of the correlation between the final BCVA and categorical variables before reoperation was determined using the Mann–Whitney U test. The significance of the correlation between final BCVA and continuous variables before reoperation was determined using Spearman’s non-parametric correlation test. The difference between the preoperative BCVA and final BCVA was determined using a paired t-test. Statistical significance was set at *P* < 0.05.

## Results

### Baseline characteristics and association between variables and final BCVA

The patients’ ages ranged from 22 to 77 years (47 ± 14 years). The axial lengths ranged from 23.38 mm to 33.11 mm (26.01 ± 2.19 mm). Ten eyes (50%) had an axial length > 26 mm. The preoperative IOP was 8–21 mmHg (13.1 ± 3.4 mmHg), and the preoperative BCVA was 2.0 LogMAR–0LogMAR (1.2 ± 0.6 LogMAR, Snellen 20/160 ± 20/63).

The interval between the last attachment surgery and re-detachment was 1 day to 2 years and 4 months, with a mean interval of 114 ± 202 days and a median interval of 28 days. The interval between the Re-RD and previous surgery was ≤ 60 days in 12 patients (60%). The duration of symptoms ranged from 1 to 30 days, with a mean of 10 ± 10 days and median of 5 days.

**Fig. 1:**
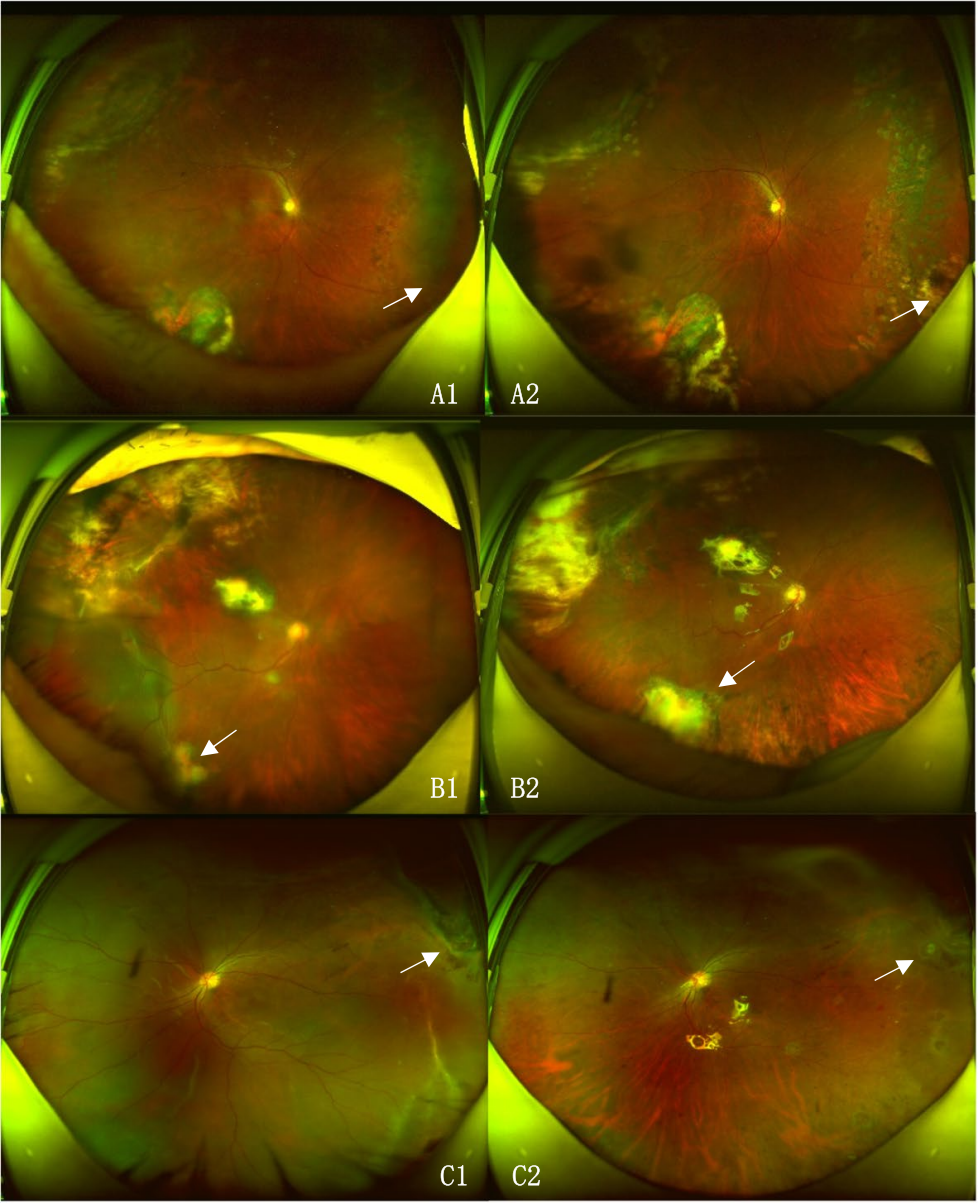
The ultra-wide-field fundus images before and after reoperations of recurrent retinal detachment patients with no break detected preoperatively. **A**, **B**, **C** were three different patients. The left images were shot when recurrent retinal detachment occurred with no break founded under thorough fundus examination. The arrows indicated the site of the tiny breaks confirmed intraoperatively which were located beside the original pigment laser scar. The pictures on the right were photographed after surgery with arrows showing the location of the tiny hole and the laser spots around

No break was detected in 12 eyes, one break in six eyes, two breaks in one eye, and four breaks in one eye before this reattachment surgery. Among the 12 eyes with no break detected preoperatively, 11 (92%) were found to have a small crevice-like break beside the pigment scar of a large number of original laser spots during surgery (Fig. 1). Anterior PVR accounted for 75% (6/8) of the patients with PVR ≥ C.

The associations between the variables before reoperation and the final BCVA are summarized in Table 1. Patients with phakic eyes, those undergoing only one surgery, and those with more than one break had better final BCVA. A significant negative correlation was found between the final BCVA and axial length (*r* = -0.6, *P* = 0.005). A significant positive correlation was also found between the final BCVA and BCVA before reoperation (*r* = 0.587, *P* = 0.007). The final BCVA was 0.6 ± 0.4LogMAR in macular-on eyes and 0.8 ± 0.8LogMAR in macular-off eyes, but the difference was not significant.

**Table 1:** Relationship between factors before surgery for Re-RD and the final BCVA

Variables		Eyes (n, %)/ mean ± SD	BCVA(LogMAR) (mean ± SD)	*P* value^#^
Sex	Men	12 (60%)	0.7 ± 0.5	0.668
	Women	8 (40%)	1.0 ± 0.9	
Laterality	Right	13 (65%)	0.8 ± 0.8	0.841
	Left	7 (35%)	0.7 ± 0.5	
Lens status^a^	1	12 (60%)	1.0 ± 0.7	0.019
	2	8 (40%)	0.4 ± 0.3	
Number of past retinal surgery	1	5 (75%)	0.3 ± 0.3	0.027
	≥ 2	15 (75%)	0.9 ± 0.7	
Previous tamponade	SO	13 (65%)	0.8 ± 0.4	0.109
	Gas	7 (35%)	0.8 ± 1.0	
Macular detachment	Yes	14 (70%)	0.8 ± 0.8	0.739
	No	6 (30%)	0.6 ± 0.4	
Inferior quadrant detachment	Yes	19 (95%)	0.7 ± 0.7	0.188
	No	1 (5%)	1.3	
Quadrants of detachment	1	5 (25%)	0.7 ± 0.5	0.825
	≥ 2	15 (75%)	0.8 ± 0.7	
PVR grading	A + B	12 (60%)	0.6 ± 0.4	0.138
	C + D	8 (40%)	1.1 ± 0.9	
Number of retinal breaks	1	16 (80%)	0.9 ± 0.7	0.05
	≥ 2	4 (20%)	0.3 ± 0.3	
Age (years)		47 ± 14		0.192
Duration of symptoms (days)		10 ± 10		0.172
Interval between Re-RD and last attachment surgery (days)		114 ± 202		0.498
Axial length (mm)		26.02 ± 2.19		0.005(*r* = -0.6)
Preoperative IOP (mmHg)		13.1 ± 3.4		0.698
Preoperative BCVA (LogMAR)		1.2 ± 0.6		0.007(*r* = 0.587)

*SO* silicon oil, *PVR* proliferative vitreoretinopathy, *IOP* intraocular pressure, *BCVA* Best corrected visual acuity

^a^Lens status: 1 = Pseudophakia or aphakia, 2 = Phakia; ^#^P value: Mann–Whitney test for categorical variables; Spearman’s non-parametric correlation test for continuous variables

### Retinal anatomical success and BCVA change

At the end of the re-vitrectomy, 17 eyes (85%) were filled with SO, and the remaining three eyes were filled with gas. The postoperative follow-up time was 3–43 months, with an average of 20 ± 13 months. At the last follow-up, three eyes were SO-dependent, of which one eye had inferior retinal detachment and two had complete retinal attachment under the SO. For the other 17 post-vitrectomy eyes, two eyes had Re-RD after SO removal, one eye gave up treatment, and the other one had complete anatomical success after another vitrectomy with gas tamponade. The remaining 15 eyes were completely attached after a single operation. The single-operation complete retinal attachment rate was 75% (15/20, 12 eyes treated with SO and later SO removal + 3 eyes treated with gas tamponade), the complete retinal attachment rate was 80% (16/20), the incomplete retinal attachment rate was 90% (18/20), and the macular attachment rate was 95% (19/20) (Table 2).

**Table 2:** Retinal reattachment rate and comparison of final BCVA and pre-reoperative BCVA

Anatomical outcome(n,%)	Final BCVA (x ± SD) LogMAR/Decimal	Preoperative BCVA (x ± SD) LogMAR/Decimal	*P* value^*^
All patients (*n* = 20,100%)	0.8 ± 0.7LogMAR (0.15 ± 0.2)	1.2 ± 0.6 LogMAR (0.06 ± 0.25)	0.008
Macular attached (*n* = 19,95%)	0.7 ± 0.4 LogMAR (0.2 ± 0.4)	1.1 ± 0.6 LogMAR (0.08 ± 0.25)	0.001
Incomplete retinal attachment (*n* = 18,90%)	0.6 ± 0.4 LogMAR (0.25 ± 0.4)	1.1 ± 0.6 LogMAR (0.08 ± 0.25)	0.001
Complete retinal attachment (*n* = 16,80%)	0.6 ± 0.4 LogMAR (0.25 ± 0.4)	1.0 ± 0.5 LogMAR (0.1 ± 0.3)	0.004
Single-operation complete retinal attachment (*n* = 15,75%)	0.5 ± 0.4 LogMAR (0.3 ± 0.4)	1.0 ± 0.5 LogMAR (0.1 ± 0.3)	0.003

^*^statistical analysis by paired-t test

**Fig. 2:**
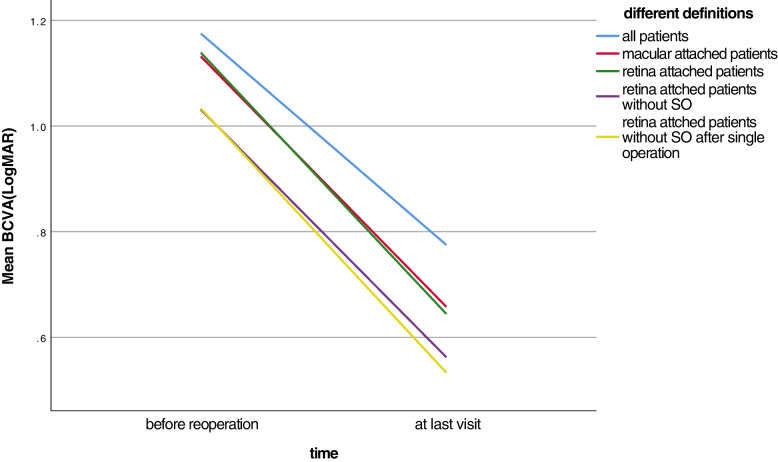
Visual acuity change before and after reoperation under different definitions. Postoperative BCVA improved significantly under different retinal status

The mean BCVA of 20 patients improved from 1.2 ± 0.6LogMAR (0.06 ± 0.25) preoperatively to 0.8 ± 0.7LogMAR (0.15 ± 0.2) at the last follow-up (*P* = 0.008) (Table 2, Fig. 2). Visual acuity under other retinal status definitions also improved significantly compared to that before surgery. At the last visit, 3 eyes (15%) had BCVA of 0LogMAR (1.0), 9 eyes (45%) had BCVA ≥ 0.5LogMAR (0.3), 3 eyes (15%) had BCVA > 0.3LogMAR (0.5), and 5 eyes (25%) has visual acuity < 1.0LogMAR (0.1).

### Postoperative complications and management

Mild elevation of IOP was observed in five SO-filled eyes. Three cases were reduced to normal IOP by anti-glaucoma drugs during SO filling, among which two eyes stopped using anti-glaucoma drugs after SO removal, and the IOP remained within the normal range; one eye was SO-dependent, and the IOP was maintained within the normal range by one anti-glaucoma eye drop. In the other two cases, IOP was still high after using anti-glaucoma eye drops, cyclophotocoagulation was combined with SO removal, and IOP dropped to the normal range afterwards. The patient who had Re-RD after SO removal and discontinued further surgical treatment had a low IOP of 6 mmHg.

All eight phakic eyes developed cataracts of varying degrees during the follow-up period. In the later stage of SO removal, seven eyes were treated with phacoemulsification extraction and intraocular lens implantation, and the other eye (Fig. 1 C1 and C2) was not treated with cataract surgery because of the young age.

One patient in this group had systemic diabetes, and due to repeated surgeries, keratitis occurred after revitrectomy, which was cured after ocular drug treatment.

### Contralateral eye condition

In the fellow eye, two eyes had already undergone PPV for RRD and one eye was also diagnosed with RRD when the treated eye was first diagnosed with RRD. Eyesight threatening disease in the fellow eye accounted for 15% of cases. During the follow-up of the other 17 eyes that were initially normal, one of them had optic nerve atrophy due to blunt trauma, with no light perception. That is, during the follow-up period of 3–43 months (mean, 20 months), 5.9% of the contralateral eyes had vision-damaging lesions.

## Discussion

We found that the single-operation complete retinal reattachment rate was 75% (15/20), the complete retinal reattachment rate was 80% (16/20), and the incomplete retinal reattachment rate was 90% (18/20) for re-vitrectomy for Re-RD in post-vitrectomy eyes. Previous studies have reported that the success rate of resurgery for Re-RD is 60%–95.2% [5, 10, 13, 19–21]. The reasons for the great disparity among previous reports are not only due to differences in population and surgical methods but also because of differences in the definition of anatomical success and follow-up time. For example, the reattachment rate of 82.1% reported by Deaner et al. [10] referred to anatomical reattachment under SO with a single operation after 1 year of follow-up. After SO removal, the complete reattachment rate for a single surgery was 57.1%. The reattachment rates of 90.4% and 95.2% reported by Mancino et al. [21] and Tatsumi et al. [13], respectively, did not determine whether SO was removed. Wei et al. [20] showed that retinal reattachment was achieved in 72.2% of eyes without tamponade in the vitreous cavity. Our research on a specific group of post-vitrectomy eyes achieved similar anatomical success rates under various anatomical success definitions of revitrectomy.

After treatment, the mean BCVA of patients in this group improved from 1.2 ± 0.6LogMAR (0.06 ± 0.25) before surgery to 0.8 ± 0.7LogMAR (0.15 ± 0.2) at the last follow-up. The BCVA of 16 patients with complete retinal reattachment improved from 1.0 ± 0.5LogMAR (0.1 ± 0.3) to 0.6 ± 0.4LogMAR (0.25 ± 0.4). At the last visit, 45% of patients had visual acuity ≥ 0.3, 15% had visual acuity > 0.5, and 25% had visual acuity < 0.1. Ender et al. [22] reported that 14.4% of patients with Re-RD recovered their visual acuity above 0.5 after treatment, which is similar to our report. However, in the study by Ambiya et al. [5], only 5.9% of patients achieved a BCVA ≥ 0.3 after re-surgery. Ambiya et al. [5] found that 65.25% of patients had a visual acuity < 0.1, and 28.81% had a visual acuity between 0.1 and 0.3 after surgery. Only 25% of our patients had visual acuity < 0.1 at the final follow-up, which may be related to the fact that all the patients in this study received timely treatment within one month after recurrence, and the proportion of visual acuity < 0.1 at onset was 40%. Previous studies have found that poor baseline visual acuity, delayed treatment, multiple previous operations, macular-off retinal detachment, and PVR ≥ C grade are important factors affecting visual acuity improvement [5, 23–26]. Our results showed that the final BCVA was positively correlated with the BCVA at recurrence and negatively correlated with axial length. Patients who underwent only one retinal surgery had a better final BCVA. Although eyes with macular on and PVR grade ≥ C had better vision than those with macular off and PVR grade ≤ B, the difference was not statistically significant. This may be because of the small sample size.

PVR is not only an important factor affecting vision restoration but also the cause of early postoperative Re-RD [2, 27]. Relevant factors for PVR formation include preoperative chronic inflammation, preoperative hypotony, inadequate posterior vitreous detachment, incomplete shaving of the vitreous base, excess retinal photocoagulation or cryopexy, retinectomy, disturbance to the intraocular environment by surgery itself, multiple intraocular surgery, postoperative vitreous hemorrhage, postoperative uveitis, and choroidal detachment [5, 8, 28–30]. All these factors lead to the spread of retinal pigment cells and destruction of the blood-eye barrier, aggravate postoperative inflammation, and stimulate the formation of PVR [30, 31]. Aaberg et al. [32] found that 86% of patients with Re-RD after vitrectomy had anterior PVR, and patients with anterior PVR generally had a worse prognosis than those with posterior PVR [33, 34]. In this study, the proportion of anterior PVR in patients with PVR-C was 75% before the reoperation. We performed a complete posterior vitreous detachment and removed the vitreous cortex as cleanly as possible. Retinectomy and retinal cryopexy were not performed. Most patients were filled with SO at the end of surgery to reduce vitreous hemorrhage, postoperative uveitis, and choroidal detachment. These details played an important role in the anatomical and visual success of our study.

With respect to our observation, in patients with no definite retinal break detected preoperatively, 92% of the retinal breaks turned out to be small crevice-like changes alongside the original excessive laser pigment scar. Previous studies on the analysis of breaks mainly focused on the reasons for not detecting breaks, causes of reopening of the primary break, and factors influencing the formation of new breaks [23–25, 35–42], but there are no relevant reports on the characteristics and location of the breaks in post-vitrectomy eyes with Re-RD when no breaks could be discovered before re-surgery. Another patient feature in our study was that Re-RD eyes tended to have high myopia, with a proportion of 51.5% eyes with axis length > 26 mm, while the incidence of high myopia in the normal Asian population is 0.8%–9.1% [43]. Scholda et al. [44] and Teke et al. [28] also found that patients with high myopia were prone to Re-RD after SO removal.

In this study, complications after revitrectomy combined with tamponade were secondary glaucoma in 25% of eyes, complicated cataract in 100% of eyes, SO dependence in 15% of eyes, keratitis in 5% of eyes, and Re-RD in 10% of eyes. In previous reports, the percentage of complicated cataract was 69%–100% [8, 38], secondary glaucoma was 30%–60.6% [3, 38, 45], SO-dependent eye was 19.7%–50% [10, 19, 38, 46], Re-RD was 30.3% [38], and SO emulsification was 11.7% [14]. Choudhary et al. [3] found that although IOP increased in 60.6% of patients before SO removal, only 7.5% of patients needed cyclophotocoagulation to reduce the IOP to the normal range after SO removal. Al-Wadani et al. [8] found that 15.7% of patients still had intraocular hypertension after SO removal, and except for 4.3% of patients who needed anti-glaucoma surgery, the IOP returned to the normal range after using anti-glaucoma eye drops. For five intraocular hypertension in our study, the IOP dropped to normal in two eyes after SO removal, the IOP was reduced to normal in one eye after administering anti-glaucoma eye drops, and the IOP returned to normal after SO removal combined with cyclophotocoagulation in two eyes (10%). The prognosis was similar to that of patients with high IOP in previous studies.

Schwartz et al. [47] found that 31.2% of the contralateral eyes of patients with PVR had vision-threatening lesions, most of which were retinal break-related, and 22% of the contralateral eyes were likely to have vision-threatening lesions within 10 years, such as RRD, age-related macular degeneration, central retinal vein obstruction, optic nerve atrophy, macular hole, myopic degenerative diseases, and glaucoma. Schimidt et al. [48] found that in patients with RRD, the probability of RRD occurring in the fellow eye within six years was 7.1%, of which 3.5% occurred within one year. In this group, three (15%) patients with Re-RD in post-vitrectomy eyes had vision-threatening disease in the contralateral eye, all of which were RRD. For the other 17 normal fellow eyes at first, traumatic optic atrophy, leading to no light perception, occurred in one eye during follow-up; that is, the ratio of eyesight-damaging lesions was 5.9% over the course of an average of 20 months.

This study has several limitations. Firstly, the number of patients was relatively small. We included only Re-RD cases in post-vitrectomy eyes operated by one surgeon using revitrectomy without retinectomy or scleral buckling. We only included patients with an initial diagnosis of RRD. Therefore, the target group of patients was relatively difficult to collect. Secondly, because some patients were not admitted to our hospital at the time of the first onset, the ocular characteristics of patients diagnosed with primary RRD before the first operation cannot be fully collected, and the characteristics of the eyes at first cannot be compared with those at the time of recurrence. Thirdly, the surgeries were performed by a single surgeon, which generated performance bias. A prospective study with more cases from multiple centers is necessary. Fourthly, one patient who gave up further treatment after Re-RD again had a vision of no light perception at the last follow up. No light perception is not actually a visual acuity measurement, but we have assigned it a value which may reduce the mean post-operative visual acuity artefactually.

## Conclusions

We found that revitrectomy without retinectomy or scleral buckling could effectively treat Re-RD in post-vitrectomy eyes. In Re-RD patients with no definite retinal break detected preoperatively, the retinal hole usually shows small crevice-like changes alongside a large number of original laser pigment scars; therefore, attention should be paid to the amount of photocoagulation. Patients with Re-RD often have vision-threatening lesions in the contralateral eye; therefore, it is important to improve the success rate of reoperations in the affected eye.

## Data Availability

The datasets used and/or analyzed during the current study are available from the corresponding author on reasonable request.

## Abbreviations

Re-RD: Recurrent retinal detachment
RRD: Rhegmatogenous retinal detachment
BCVA: Best corrected visual acuity
PVR: Proliferative vitreoretinopathy
PPV: Pars plana vitrectomy
SO: Silicon oil
IOP: Intraocular pressure
